# Primary and secondary three-month outcomes of a cluster-randomized trial of home-based postpartum contraceptive delivery in southwest Trifinio, Guatemala

**DOI:** 10.1186/s12978-020-00974-z

**Published:** 2020-08-20

**Authors:** Margo S. Harrison, Saskia Bunge-Montes, Claudia Rivera, Andrea Jimenez-Zambrano, Gretchen Heinrichs, Antonio Bolanos, Edwin Asturias, Stephen Berman, Jeanelle Sheeder

**Affiliations:** 1 University of Colorado, Anschutz Medical Campus, Mail Stop B198-2, Academic Office 1, 12631 E. 17th Avenue, Rm 4211, Aurora, CO 80045 USA; 2 Fundación para la Salud Integral de los Guatemaltecos (FSIG), Quetzaltenango, Guatemala; 3 Denver Health, Denver, CO USA

**Keywords:** Postpartum contraception, Implant, Guatemala

## Abstract

**Design:**

This a cluster-randomized parallel arm pragmatic trial to observe the association of home-based postpartum contraceptive provision, including the contraceptive implant, with implant utilization rates at 3 months post-enrollment.

**Methods:**

In a region of rural Guatemala referred to as the Southwest Trifinio, twelve communities are served by a community-based antenatal and postnatal care program. The communities were combined into eight clusters based on 2017 birth rates and randomized to receive the home-based contraceptive delivery (condoms, pills, injection, implant) during the routine 40-day postpartum visit. All participants receive comprehensive contraceptive counseling beginning at the first antenatal visit, so control clusters received this as part of routine care; this education preceded the study intervention.

**Results:**

Once the 12 communities were combined into 8 clusters by expected birth volume and nurse team, which we expected to translate to eventual postpartum visits, the allocation sequence was generated in SAS. Of 208 women enrolled in the study, 108 were in four intervention and 100 in four control clusters. We used descriptive statistics to produce counts and percentages of characteristics of the study population overall and by intervention arm followed by univariate modeling using a mixed effects regression adjusted for cluster. Three-month contraceptive initiation rates were 56.0% in the control clusters compared to 76.8% in the intervention clusters, *p* < 0.001. Women in control clusters overwhelmingly opted for the injectable contraceptive (94.6%) while women in intervention clusters chose both the injection (61.5%) and the implant (33.7%), *p* < 0.001. Implant use by 3 months, the primary outcome of the study, was significantly higher in the intervention arm (25.9%) compared to the control arm (3.6%), *p* < 0.001, RR 1.3 CI [1.2, 1.4].

**Conclusion:**

Our study was designed to respond to previously identified barriers to contraceptive uptake, and it was successful. Not only did it increase overall use of contraception by 3 months, but it shifted that contraceptive use away from short-acting methods in favor of longer-acting methods, with high continuation and satisfaction rates and no adverse outcomes reported.

**Trial registration:**

clinicaltrials.gov, NCT04005391; Retrospectively Registered 7/2/2019,

## Plain English summary

Postpartum contraception is important for spacing and preventing undesired or unplanned pregnancies. In Guatemala, two out of every three women have an unmet need for contraception. In a rural area in the Southwest of the country, there is a region that is home to a large migrant worker community. The community is served by home-based community nursing programs that provide maternal and child health. Preliminary data from the community suggests that most women’s need for postpartum contraception is met through the community maternal health program, but primarily through short-acting contraceptive methods; there are access barriers to the use of long-acting contraceptives. We designed a trial that randomized subgroups of the community into two arms; one that received a home-based postpartum contraceptive intervention where nurses brought modern contraceptives (including the long-acting implant) to women’s forty-day postpartum visit, and the other arm received standard postpartum care, which included postpartum contraceptive education, but not provision of the methods. When we surveyed the women who consented to participate in the trial at 3 months, we found that the rate of contraceptive uptake in the intervention communities was higher overall than in the control communities, and that the rate of implant use was significantly higher as well. Our study was designed to respond to previously identified barriers to contraceptive uptake, and it was successful; it shifted that contraceptive use away from short-acting methods in favor of longer-acting methods, with high continuation and satisfaction rates and no adverse outcomes reported.

## Introduction

Postpartum contraception is important to prevent unintended, undesired, and closely-spaced pregnancies [1]. In Latin America, 66% of reproductive age women do not desire pregnancy, but over a fifth of them are not using effective contraception, and end up accounting for 75% of unintended pregnancies in the region [2]. Prior research has found an estimated a 67.6% unmet need for postpartum contraception in Guatemala, with only 25.8% of the population utilizing modern contraceptives postpartum [3]. In our study population of interest in the Southwest Trifinio, analysis of historical unpublished data suggests that about 88% of women in the region are using or are interested in using contraception by 40 days postpartum. Of these users, 0.5% used condoms, 0.5% pills, 0.5% lactational amenorrhea, 1.5% natural family planning, almost 4% long-acting reversible contraceptives (around 3% using the implant), 21% sterilization, and 72% opted for injectable contraception. According to the literature, if women receive comprehensive contraceptive education, around 11% will choose to use the contraceptive implant [4]. Prior research from our community has found barriers to the use of long-acting reversible contraceptives (LARC) include lack of spousal approval, difficulty accessing contraceptive methods, lack of knowledge, and fear of adverse effects [5]. Therefore, we hypothesized that if we addressed difficulty accessing contraceptive methods by bringing contraceptives to women’s homes, we would increase our implant uptake rate.

Our study, the protocol of which has been published, was a cluster-randomized, pragmatic, parallel arm trial whereby women in intervention clusters had experienced study nurses bring contraceptives (condoms, pills, injection, implant) to postpartum mothers’ in their homes [6]. The objective of the study was to observe if the reduced access barrier by providing contraceptives at the final home visit was associated with an increase in implant uptake by 3 months postpartum. The objective and hypothesis pertain to the individual participant level. Our pre-specified primary outcome was implant initiation among women by 3 months post-enrollment, by study arm [6]. Our pre-specified secondary outcome measures were overall contraceptive uptake among women at three and 12 months post-enrollment as well as contraceptive continuation, satisfaction, and pregnancy rates, by study arm [6]. These outcomes pertain to the cluster and participant level.

## Methods

### Setting

The University of Colorado, through a partnership with AgroAmerica, supports a clinic and community-based maternal and child health programming in a rural area of Guatemala that borders Mexico referred to as the Southwest Trifinio region [7]. The maternal health program, referred to as Madres Sanas, currently provides four antenatal and two postnatal visits to women in their homes [7]. The program, which began in 2011, has prioritized contraceptive education prior to study initiation, in order to address the barriers of lack of knowledge and fear of adverse effects. Our nurses already provided education to all women in the program on contraception starting at the first prenatal visit [7].

### Trial design

**Fig. 1:**
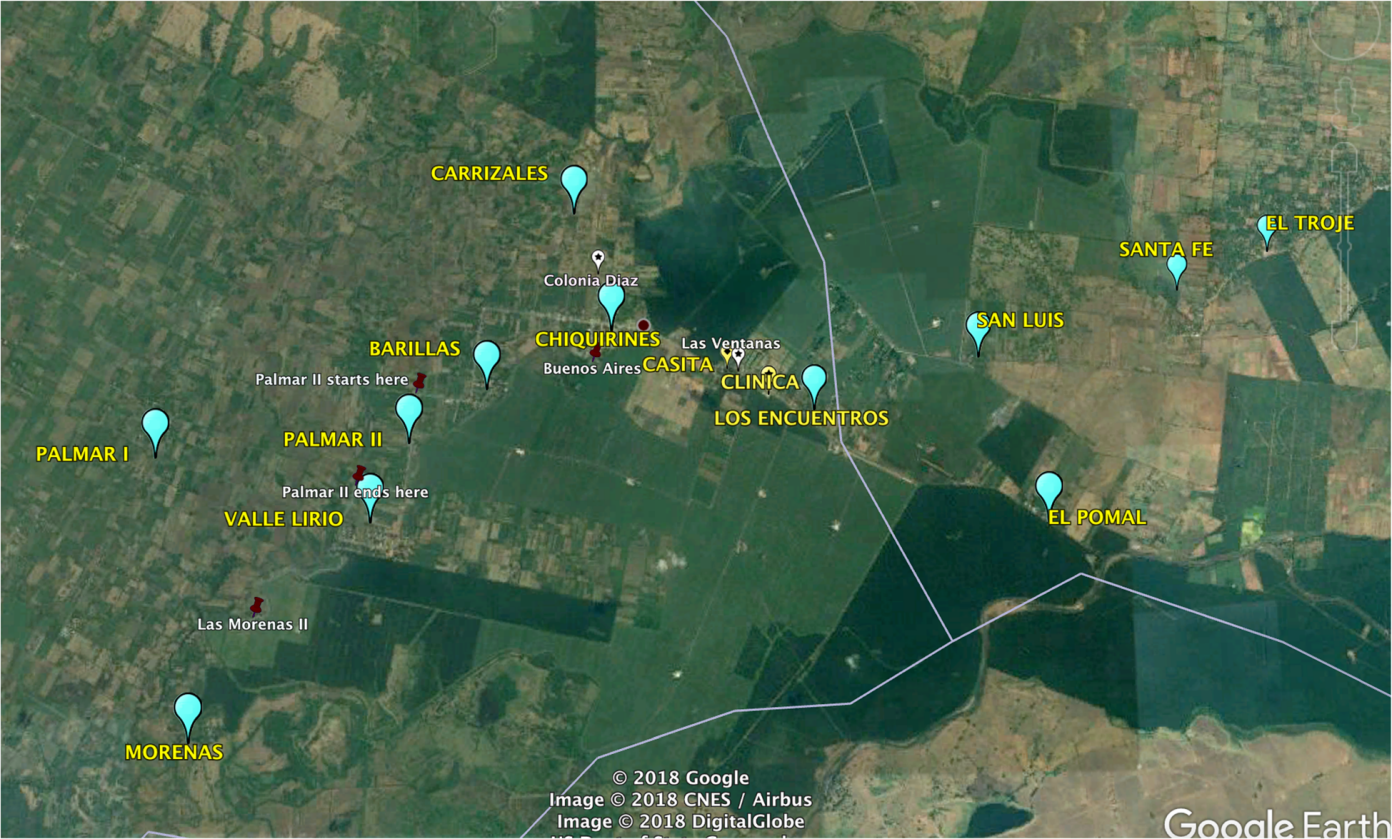
Map of the Southwest Trifinio Region and Communities

Our study, the protocol of which has been published, was a cluster-randomized, pragmatic, parallel arm trial [6]. The rationale for using a cluster design was that the region is already historically divided into twelve communities that define themselves by their cultural identity (Fig. 1). Nurses are assigned to communities and provide care to women through that organizational structure, and as such, it would likely have been less successful to divide the region into clusters through another mechanism, and an individual-randomized design would not have respected the correlation that exists within these communities. The twelve communities in the Southwest Trifinio were combined into eight clusters based on data from births by community in 2017 [6]. The clusters combined communities already served by the Madres Sanas nurse teams with around twenty-five expected births (postpartum visits) per cluster over the year of projected study enrollment [6]. Clusters were then randomized to intervention or control status, within each nurse team (Table 1) [6]. There were no changes to these methods after trial commencement.

**Table 1:**
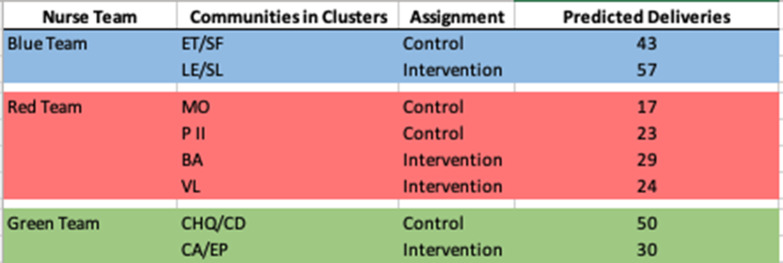
Cluster Randomization by Nurse Team.

### Randomization/blinding

Once the clusters were assigned by expected birth volume, which we expected to translate to eventual postpartum visits, the allocation sequence was generated [6]. The initial allocation sequence was generated using SAS to assign the clusters to either the intervention or the control arm of the trial [6]. Once the nurses were educated about the study and understood all study procedures and activities, they were informed about the cluster assignment [6]. One of the nurse teams was unenthused about participation given they were not assigned an intervention group; as a result the nursing supervisor requested that the randomization be rerun and each nurse team have an intervention and a control group [6]. As such, the allocation sequence was rerun to accommodate the “real world” constraints of the study to respond to the request of the study staff in order to proceed with study activities [6]. How the communities were combined into clusters and assigned to nurse teams, as well as study arm can be found in the published protocol [6]. Allocation was therefore at the cluster level and was not concealed; the protocol also did not involve any blinding of study staff or participants [6]. When a woman was visited for her postpartum visit, whether or not she was offered the intervention depended on the community in which she resided [6].

### Interventions/implementation

The forty-day postpartum visit is a routinely scheduled visit as part of the Madres Sanas program [7]. Culturally, women tend to remain abstinent for 40 days after birth, which is why the visit is scheduled at that time [8]. Nurse teams visited women in their homes for the postpartum visit and provided routine postpartum care per World Health Organization guidelines [7, 9]. After the provision of routine care, women were screened, offered enrollment, and consented to participate in the trial [6]. For women in control clusters, they consented to be followed for a year for study staff to observe their contraceptive and reproductive decisions. For women in the intervention arm, they consented to be offered contraceptives in their home followed by a year of observation of their contraceptive and reproductive decisions. Routine postpartum care was provided to women in control and intervention clusters (this included comprehensive contraceptive education through the program to all women) [6].

For intervention clusters, nurses brought a contraceptive kit to the visit [6]. The kit was stocked with 10 condoms (Vive Amor®), one pack of pills (Segura Plus®), one syringe of medroxyprogesterone (Cyclofem®), and one implant (Jadelle®), as well as all the necessary materials to place the implant or administer the injection under sterile conditions [6]. Contraceptives were financed through the study at no cost to participants and were procured from local providers, as all methods are routinely available in Guatemala. However, outside the context of the study, the closest location to get an implant is in Coatepeque, Guatemala, which is about an hour away from the site [6]. Prior to offering these methods, patients were screened using Medical Eligibility Criteria to ensure safety [6, 10]. The intervention was administered by cluster, at the individual participant level.

### Participants

Women were eligible to enroll if they were between the ages of 15–35, and they had not already started a contraceptive method by the time of study enrollment (the forty-day postpartum visit) [6]. There were no eligibility criteria for clusters. All communities were offered enrollment when the study was discussed with the Community Advisory Board, and all communities agreed to participate, resulting in the participation of all clusters. The setting in which data was collected was in the home setting of women who were seen as part of the Madres Sanas program [6, 7]. Those women who had received antepartum care through the program, delivered, and were being evaluated for their routinely scheduled forty-day postpartum visit were offered enrollment [6]. The eligibility screen, offer of participation, and consent process took place in the woman’s home after routine forty-day postpartum care was provided [6]. Adolescents were able to assent with consent of an adult spouse or parent [6].

### Sample size

Based on historical data from the Madres Sanas program, we expected over the course of the year of projected enrollment to observe around 260 women who would meet eligibility criteria [6]. To detect a change in implant uptake rates from 3 to 15% at 85% power with 5% significance and an intraclass correlation of 2%, with equal cluster sizes assumed (25 births), our sample size was calculated to require 200 women, 100 in each arm [6]. As this was a pragmatic trial testing a hypothesis about how reducing a barrier to access might increase use of already approved and commercially available contraceptives, there were no pre-specified stopping guidelines [6].

### Outcomes

Our pre-specified primary outcome was implant initiation among women by 3 months post-enrollment, by study arm [6]. Our pre-specified secondary outcome measures were overall contraceptive uptake among women at three and 12 months post-enrollment as well as contraceptive continuation, satisfaction, and pregnancy rates, by study arm [6]. These outcomes pertain to the cluster and participant level. After the trial commenced, we did add on additional secondary outcomes. We wished to collect implementation outcomes data on the reach, effectiveness, adoption, and implementation of our study intervention. These outcomes were assessed by conducting a nurse survey and focus group at the 3-month timepoint with the addition of survey questions to the twelve-month patient survey. We added these outcomes as we felt we could contribute not only to the effectiveness literature, but to the implementation literature to assist others who might wish to disseminate our findings.

### Statistical methods

We used descriptive statistics to produce counts and percentages of characteristics of the study population overall and by intervention arm. We performed univariate comparisons with a mixed effects regression adjusted for cluster of these characteristics to ensure randomization was effective, which it was; *p*-values were not significant and not shown. We also performed comparisons of implant use at 3 months (primary outcome) and overall method use at 3 months (secondary outcome) by intervention arm, using a mixed effects regression adjusted for cluster that converted the comparisons to risk ratios with 95% confidence intervals. We continued to use descriptive statistics to produce counts and percentages of contraceptive uptake and use by intervention arm and by study timepoints, describing initial method choices in the intervention clusters as well as use of methods by 3 months in all study participants. These same methods were used to describe secondary outcomes of continuation and satisfaction and reasons for contraceptive choices among study participants. STATA software version 15.2 (StataCorp LP, College Station, TX, USA) was used for analysis.

## Results

**Fig. 2:**
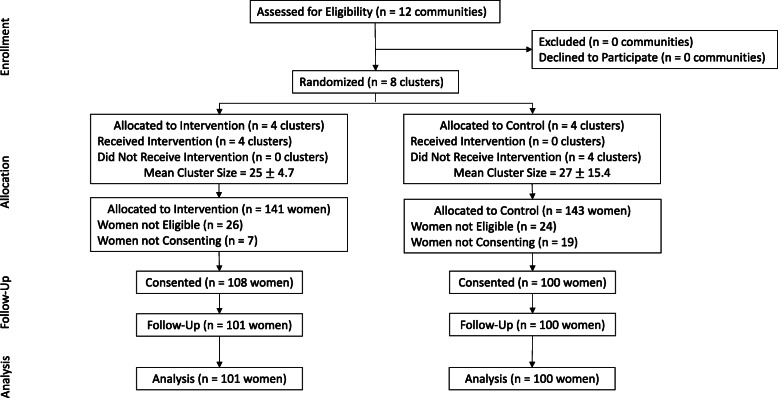
CONSORT DIAGRAM

Fig. 1 is a map of the Southwest Trifinio communities included in the trial, and Table 1 illustrates how the communities were clustered and randomized by nurse team. Figure 2 presents the participant flow through the trial. There were no eligibility criteria for communities and all community leaders agreed to support participation in the study with none excluded. Using previously described methods these communities were matched on expected births, by nursing teams, into eight clusters that were then randomized in order that each nurse team had at least one intervention and control group. Four clusters were randomized to intervention, four to control. All intervention clusters received the intervention with no control clusters receiving the intervention, as planned. Enrollment began October 10, 2018 and ceased September 24, 2019. The mean size of intervention clusters was 25 women with a 4.7 standard deviation. The mean cluster size of control clusters was 27 women with a 15.4 standard deviation. Of the 141 women approached to participate in the study in the intervention clusters, 26 were not eligible and 7 declined to participate. Of 108 women who received the intervention, 3-month outcome data was available for 101 of them. In the control clusters, of 143 women approached for study participation 24 were not eligible and 19 declined to consent. Of the 100 women who were enrolled, none were lost to follow-up by 3 months.

Table 2 describes the overall study population and the subpopulations of women by intervention arm. Women were young, with a median age around 22 years old, the majority had had some education (around 90%), and most were married (around 88%). The largest subpopulation of the cohort was primiparous (around 39%) and the majority had received greater than four prenatal visits (about 81%) through the Madres Sanas program. About two-thirds of women experienced vaginal birth and were delivered by a nurse or physician, about half delivered in a facility, and around three-fourths gave birth to infant weighing at least 2500 g. The majority of babies were born alive and remained alive by the 72-h postpartum visit (around 93%), most were alive at the 40-day enrollment visit (about 95%), around 7% of women were already sexually active by the time of enrollment, and over one-third of women did not desire future fertility (about 37%). Randomization was effective and study arms were not statistically different by any of these characteristics when checked (*p*-values not shown).

**Table 2:** Characteristics of Study Population by Cluster

	Total Population (*n* = 208)	Control Clusters [4] (*n* = 100, 48.1%)	Intervention Clusters [4] (*n* = 108, 51.9%)
*Sociodemographic Characteristics*			
Age in years (median IQR)	21.8 [18.7,25.4]	21.9 [18.8,25.4]	21.8 [18.7,25.5]
Missing	3 (1.4%)	1 (1.0%)	2 (1.9%)
Education			
None	17 (8.2%)	8 (8.0%)	9 (8.3%)
Any	188 (90.4%)	91 (91.0%)	97 (89.8%)
Missing	3 (1.4%)	1 (1.0%)	2 (1.9%)
Married			
Yes	183 (88.0%)	88 (88.0%)	95 (87.9%)
No	23 (11.1%)	12 (12.0%)	11 (10.2%)
Missing	2 (0.9%)	0 (0.0%)	2 (1.9%)
*Obstetric and Antepartum Characteristics*			
Parity			
1	81 (38.9%)	39 (39.0%)	42 (38.8%)
2	59 (28.3%)	31 (31.0%)	28 (25.9%)
3	33 (15.9%)	15 (15.0%)	18 (16.7%)
4+	33 (15.9%)	15 (15.0%)	18 (16.7%)
Missing	2 (1.0%)	0 (0.0%)	2 (1.9%)
Number of Madres Sanas Prenatal Visits			
< 4	28 (13.4%)	13 (13.0%)	15 (13.9%)
4+	169 (81.3%)	82 (82.0%)	87 (80.6%)
Missing	11 (5.3%)	5 (5.0%)	6 (5.5%)
*Delivery Characteristics*			
Mode of Delivery			
Vaginal Birth	127 (61.0%)	63 (63.0%)	64 (59.3%)
Cesarean Birth	69 (33.2%)	31 (31.0%)	38 (35.2%)
Missing	12 (5.8%)	6 (6.0%)	6 (5.5%)
Location of Delivery			
Home, Private Clinic, or Other	91 (43.7%)	42 (42.0%)	49 (45.4%)
Facility (Hospital)	106 (51.0%)	53 (53.0%)	53 (49.1%)
Missing	11 (5.3%)	5 (5.0%)	6 (5.5%)
Birth Attendant			
Comadrona (TBA, “unskilled”)	61 (29.3%)	29 (29.0%)	32 (29.6%)
Nurse or Physician (“skilled”)	135 (64.9%)	64 (64.0%)	71 (65.7%)
Missing or “I don’t know”	12 (5.8%)	7 (7.0%)	5 (4.6%)
Birthweight at Delivery			
≤ 2500 g	28 (13.5%)	10 (10.0%)	18 (16.7%)
2500 g+	161 (77.4%)	81 (81.0%)	80 (74.0%)
Missing	19 (9.1%)	9 (9.0%)	10 (9.3%)
*Postpartum Characteristics*			
Sex of Infant			
Male	92 (44.2%)	40 (40.0%)	52 (48.2%)
Female	101 (48.6%)	53 (53.0%)	48 (44.4%)
Missing	15 (7.2%)	7 (7.0%)	8 (7.4%)
Infant Outcome			
Macerated Stillbirth	0 (0.0%)	0 (0.0%)	0 (0.0%)
Fresh Stillbirth	2 (1.0%)	1 (1.0%)	1 (0.9%)
Born Alive, died before 72-h visit	2 (1.0%)	0 (0.0%)	2 (1.8%)
Born Alive, alive at 72-h visit	194 (93.3%)	94 (94.0%)	100 (92.6%)
Missing	10 (4.7%)	5 (5.0%)	5 (4.7%)
Infant Status at 40 days Postpartum			
Alive	197 (94.7%)	95 (95.0%)	102 (94.4%)
Dead	2 (1.0%)	1 (1.0%)	1 (0.9%)
Missing	9 (4.3%)	4 (4.0%)	5 (4.7%)
Sexual Activity Since Birth			
Yes	16 (7.7%)	6 (6.0%)	10 (9.3%)
No	181 (87.05%)	91 (91.0%)	90 (83.3%)
Missing	11 (5.3%)	3 (3.0%)	8 (7.4%)
Desired Timeframe Until Next Pregnancy			
Approximately 2 years	1 (0.5%)	0 (0.0%)	1 (0.9%)
Approximately 3 years	8 (3.8%)	4 (4.0%)	4 (3.7%)
> 3 years	71 (34.1%)	30 (30.0%)	41 (38.0%)
I don’t know	44 (21.2%)	21 (21.0%)	23 (21.3%)
No more children desired	77 (37.0%)	42 (42.0%)	35 (32.4%)
Missing	7 (3.4%)	3 (3.0%)	4 (3.7%)

Note: no bivariate comparisons using a generalized liner model mixed effects regression adjusted for cluster were significant, pvalues not shown

Table 3 presents the primary outcome of the paper, which was comparing implant uptake at 3 months by study arm. First, however, it illustrates overall contraceptive use by 3 months and how that varies by study arm. In control clusters 56.0% of women were using a method compared to 76.8% of women in intervention clusters, *p* < 0.001, RR 1.3 [1.1,1.5]. It then shows how that usage breaks down by method of contraception including modern and traditional methods. Before this current study regarding implant usage, we observed high usage of short-acting methods, which included condoms, pills, and the injection. The use of these methods did not vary by study arm with respect to use by 3 months (although 94.6% of women in control clusters opting for these methods compared to 61.4% of women in intervention clusters), *p* = 0.72, RR 0.98 [0.8,1.1]. Finally, for our primary outcome, implant usage by 3 months was statistically significantly different between study arms with 2 women from control clusters using the method compared to 28 women in the intervention arm, *p* < 0.001, RR 1.3 [1.2,1.4]. Of note, no adverse outcomes or pregnancies have been reported to date (data not shown).

**Table 3:** Implant Utilization by Three Months (Primary Outcome) and Overall Contraceptive Use by Three Months (Secondary Outcome)

	Total Population of Women who Initiated a Method by 3 Months (*n* = 208)	Control Clusters [4] Women who Initiated a Method by 3 Months (*n* = 100, 48.1%)	Intervention Clusters [4] Women who Initiated a Method by 3 Months (*n* = 108, 51.9%)	*P*-Value	RR [95% CI]
Using a Method by 3 Months				< 0.001	1.3
Yes (% of total)	139 (66.8%)	56 (56.0%)	83 (76.8%)		[1.1, 1.5]
Method Being Used at 3 Months				< 0.001	5.6
No Method	62 (29.8%)	44 (44.0%)	18 (16.7%)		[2.6,12.0]
Abstinence	0 (0.0%)	0 (0.0%)	0 (0.0%)		
Natural Family Planning	4 (1.9%)	1 (1.0%)	3 (2.8%)		
Lactational Amenorrhea	0 (0.0%)	0 (0.0%)	0 (0.0%)		
Condoms	1 (0.5%)	0 (0.0%)	1 (0.9%)		
Pills	4 (1.9%)	0 (0.0%)	4 (3.7%)		
Injection	99 (47.6%)	53 (53.0%)	46 (42.6%)		
Implant	30 (14.4%)	2 (2.0%)	28 (25.9%)		
Intrauterine Device	0 (0.0%)	0 (0.0%)	0 (0.0%)		
Female Sterilization	1 (0.5%)	0 (0.0%)	1 (0.9%)		
Male Sterilization	0 (0.0%)	0 (0.0%)	0 (0.0%)		
Missing	7 (3.4%)	0 (0.0%)	7 (6.5%)		
Using a Short-Acting Method^a^ at 3 Months				0.72	0.98
Yes (% of users)	104 (74.8%)	53 (94.6%)	51 (61.4%)		[0.8,1.1]
Using the Implant at 3 Months				< 0.001	1.3
Yes (% of users)	30 (21.6%)	2 (3.6%)	28 (33.7%)		[1.2,1.4]

Note: *p*-values the result of a generalized linear model mixed effects regression adjusted for cluster

^a^Short Acting Method Includes: condoms, pills, injection

**Fig. 3:**
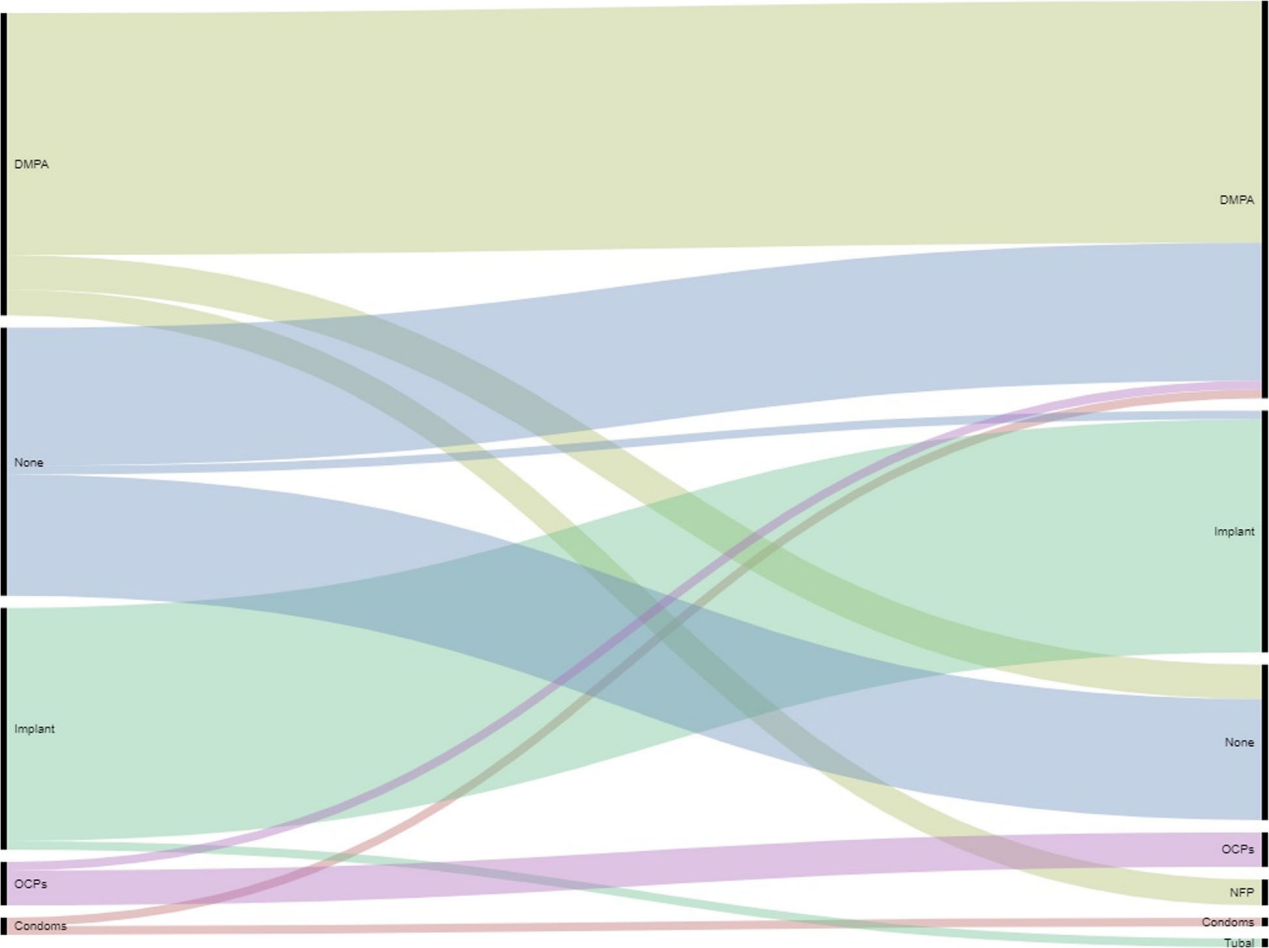
INTERVENTION CLUSTERS, Enrollment and Three-Month Contraceptive Use Among Women. NFP: Natural Family Planning, LAM: Lactational Amenorrhea, IUD: Intrauterine Device

Fig. 3 visually illustrates the contraceptive choices of women in the intervention arm of the trial who were offered free contraceptives in their homes at the time of their 40-day postpartum visit as well as the method they were using by 3 months post-enrollment. Seventy-four of the 108 women (68.5%) in the intervention clusters initiated a contraceptive method at enrollment; 2 opted for condoms (1.9%), 5 for pills (4.6%), 37 for the injection (34.3%), and 30 for the implant (27.8%). The remaining 34 women (31.5%) declined to initiate a method with no missing data at that timepoint. By the 3-month post-enrollment timepoint, the continuation rates of these methods were 50, 80, 76, and 90%, respectively. In the intervention population, by 3 months, 18 women were still not using a method (16.7%) and 7 (6.5%) had been lost to follow-up. Of those that had initiated, continued, or started a second method, 3 women (2.8%) were using natural family planning, 1 (0.9%) condoms, 4 pills (3.7%), 46 (42.6%) the injection, 28 (25.9%) the implant, and 1 (0.9%) sought female sterilization.

Table 4 presents secondary outcomes collected at 3 months in the intervention arm. Of women still on their initial method of choice, 90% were very satisfied. Of those who discontinued (*n* = 11), 3 discontinued due to side effects, 2 due to partner preference, 1 did not know why she discontinued, 2 did not want to contracept anymore, 1 sought sterilization, and 1 forgot to take her pills. Of the women who discontinued and did not initiate another method (*n* = 4), each one had a different reason, including: partner preference, could not continue due to resource constraints, did not know, and did not want to contracept anymore. In the population of women who originally declined a method at the 40 day visit (*n* = 34), 19 subsequently initiated a method by seeking it in the community, and 100% of them reported they were very satisfied on that method. Of the remaining 15 women still using no method by 3 months (44% of those who originally declined), 13 reported a reason for not initiating a method, which included: not having a partner or due to partner preference, not knowing, unable to obtain a method because the health post was closed, declining to start because menses had not resumed, not wanting to use a method, and not wanting to “ruin” her uterus.

**Table 4:** INTERVENTION ARM, Three Month Secondary Outcomes

**Satisfaction of women still on method at 3 months (*n*** **= 60)**			
Satisfaction Level on Method	Very Satisfied	54	90.0%
	A Little Satisfied	0	0.0%
	A Little Dissatisfied	5	8.3%
	Very Dissatisfied	1	1.7%
	Missing	0	0.0%
**Reasons women discontinued initial method (*n*** **= 11)**			
Reason for Initial Method Discontinuation	Did not Like Side Effects	3	27.2%
	Partner Not Permitting Contraceptive Use	2	18.2%
	Did not Know	1	9.1%
	Other: • Did not want to contracept anymore [2] • Sterilization • Forgot to take pills	4	36.4%
	Missing	1	9.1%
Reason for Not Starting Another Method (*n* = 4)	Partner Not Permitting Contraceptive Use	1	25.0%
	Doesn’t Want to Start Because Can’t Continue Due to Cost, Time, Transport	1	25.0%
	Doesn’t Know	1	25.0%
	Other: Did not want to contracept anymore	1	25.0%
**Satisfaction among women who started a method after originally declining, and** **Reasons women cited for not initiating a method among those who did not start a method by three months**			
Satisfaction Level on Method (*N* = 19)	Very Satisfied	19	100.0%
	A Little Satisfied	0	0.0%
	A Little Dissatisfied	0	0.0%
	Very Dissatisfied	0	0.0%
	Missing	0	0.0%
Reason for Not Choosing a Method (*N* = 15)	No Partner	7	46.6%
	Partner Not Permitting Contraceptive Use	1	6.7%
	Doesn’t Know	1	6.7%
	Other: • Health post was closed • Has not menstruated yet • Does not want to use a method • Does not want to “ruin” uterus	4	26.7%
	Missing	2	13.3%

**Fig. 4:**
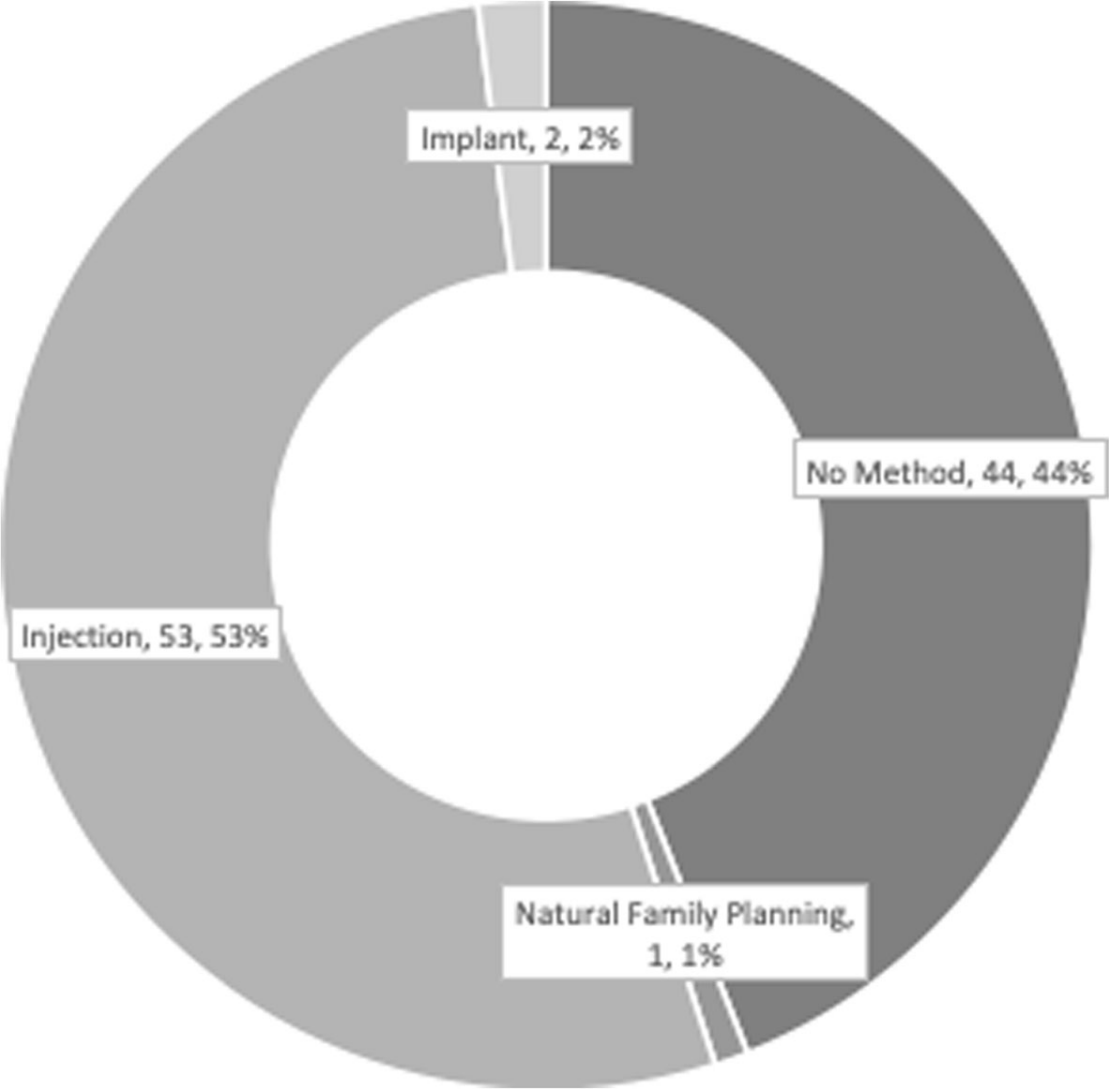
CONTROL CLUSTERS, Contraceptive Use by Three Months

Fig. 4 and Table 5 illustrate the experience of women in the control clusters. Of the 100 women followed through 3 months, with no lost to follow-up, 44 (44%) had not initiated a method. Reasons these women cited for not choosing a method were: not having a partner or due to partner preference, could not continue due to resource constraints, did not know, did not want to, wanted to menstruate first, wanted to wait until the baby was older, was already using or wanted another method (natural family planning, intrauterine device, sterilization), and forgot or was afraid to start a method. In the remaining 56 women who chose to start a contraceptive by 3 months, 53 sought the injection, 1 used natural family planning, and 1 woman obtained the implant. These 56 women were largely very satisfied or a little satisfied with the method they chose (*n* = 53), with 2 women reporting a little dissatisfaction and 1 woman reporting she was very dissatisfied.

**Table 5:** CONTROL ARM, Three Month Secondary Outcomes

Satisfaction Level on Method (*n* = 56)	Very Satisfied	48	85.7%
	A Little Satisfied	5	8.9%
	A Little Dissatisfied	2	3.6%
	Very Dissatisfied	1	1.8%
Reason for Not Choosing a Method (*n* = 44)	No Partner	9	20.4%
	Partner Not Permitting Contraceptive Use	5	11.4%
	Doesn’t Want to Start Because Can’t Continue Due to Cost, Time, Transport	1	2.3%
	Doesn’t Know	3	6.8%
	Other: • Health post was closed [2] • Spouse sick with tuberculosis • Using natural family planning [2] • Wants to be sterilized • Afraid to use contraceptives • Does not want to [2] • Wants to menstruate [2] • Forgot to start a method • Will start later, when baby is older [3] • Wants the Copper Intrauterine Device • Spouse working far away	17	38.6%
	Missing	9	20.5%

## Discussion

Our cluster-randomized parallel-arm pragmatic trial designed to test the hypothesis that reducing barriers to accessing the contraceptive implant would increase usage at 3 months, was a positive trial. By 3 months, the intervention clusters had a higher overall contraceptive uptake rate, but in the entire study population the use of postpartum contraception was 66.8%. The significantly higher rates of contraceptive use in the intervention clusters was driven by implant uptake as there was no difference in rates of short-acting modern contraceptive utilization by 3-month between the arms. In intervention clusters, continuation rates ranged from 50.0–90.0% with the highest continuation among implant users, and 90% of that population was very or a little satisfied. In control clusters, among women who initiated a method by 3 months, 94.6% of women were very or a little satisfied with their choice. The historical (2015–2017) implant use rate of around 3.0% was similar to the use rate in our control clusters, but significantly different from the use in the intervention clusters.

Observing contraceptive use patterns in the intervention cohort enhances an understanding of contraceptive seeking behavior. In terms of contraceptive initiation, almost a third of women (27.8%) opted for a long-acting reversible contraceptive (LARC) implant. Traditionally, use of LARC in Latin America and the Caribbean is low, with calls from prominent authors and organizations published in *Lancet Global Health* for LARC promotion, availability through suitable family planning services, information, and counseling in this region [11]. We feel our work has responded to that call and shown that information and counseling (provided through the Madres Sanas program pre-study) coupled with a reduced access barrier through the provision of suitable, home-based family planning services, has successfully increased LARC uptake. Therefore, our positive trial has not only potentially changed lives in our community of focus, but has also shown that this work has the potential to be disseminated more widely in the region in response to a woman’s health priority laid out by the Pan American Health Organization (PAHO) [11].

However, though initial contraceptive uptake and decision-making is important, continuing contraceptives in order to achieve family planning aims or to reduce undesired, unintended, or closely spaced pregnancies is also important [1]. Three-month continuation rates in our intervention arm are relatively consistent with data from high-income countries, with long-acting methods shown to have higher continuation rates than shorter acting methods [12]. Continuation rates seem to correlate with satisfaction rates in the literature, which is also evidenced by our findings; women in intervention clusters who continued their initial method of choice were also satisfied with that choice [12].

Decision-making regarding postpartum contraception among women in the control arm of the trial is also informative. Historically, as presented previously, women in this community were overwhelmingly using the short-acting injectable contraceptive as their primary means of postpartum contraception. Our unpublished data suggest the rate was a high as 72%. In the control clusters of our study, among the 56 women who started a method by 3 months, 53 women (94.6%) opted for the injection. This is consistent with the literature which suggests that in low- and middle-income countries, women rely overwhelmingly on short-acting methods (51–96%), which is often the injection [13]. It is very interesting that use of short-acting methods by 3 months did not vary by intervention group. Our results suggest that our intervention shifted postpartum contraceptive use in the community from the injection to the implant for those who immediately initiated a method, but for women who did not initially choose the long-acting method, the injection was still the preferred contraceptive.

The final result we wish to address is the role of a woman’s partner in decision-making regarding her contraceptive use in this setting. While women in the intervention clusters reported discontinuation due to side effects and other reasons, partner “not permitting contraceptive use” was not only a reason for discontinuation, but also a reason for not starting another method, and for not starting a method among women who initially declined a method at enrollment. Similarly, in control clusters, decision-making referenced the partner’s permission to contracept as an influence in women’s decision making (11.4% of those who did not start a method). Prior research has found overall acceptance of contraceptive use may be higher among women than men in areas of Latin America and the Caribbean with changing norms conflicting with established beliefs and practices [14]. In our community we previously found that a barrier to contraceptive use (LARC use, specifically) was lack of spousal approval, which suggests that areas for future research may include incorporating male partner involvement as a specific aim of the Madres Sanas program or considering prospective interventions to improve family planning information and counseling targeted at males [5].

Our trial, which was pragmatic in nature, may have been limited by the lack of specificity in standard operating procedures regarding counseling on contraceptives. The nurses use visual flip charts for education and the consent process, which may offer some consistency, but the fact that we did not script these activities may have allowed for variable information to be given to participants. This “fidelity” to the intervention intent will be explored as one of our implementation outcomes. Potential sources of bias include participation bias. We noted that a large number of women reported (Table 1) that they no longer desired children, or that they desired to space their pregnancies by at least two to 3 years. As we do not have this information on women who were excluded or declined to participate, this may suggest that women who participated in the trial were more interested in and likely to use postpartum contraception, which may bias our results towards higher rates of postpartum contraceptive uptake. This may reduce generalizability of our findings, although we have found that about 45–63% of women in high-income settings initiate postpartum contraception [15, 16]. Our population, however, is one of migrant agricultural workers, with diverse ethnic and cultural backgrounds, differing from the more insular Mayan populations in Guatemala, which we feel may increase the generalizability of our findings within Latin America or beyond [7].,

In conclusion, our study was designed to respond to previously identified barriers to LARC uptake, and we found that our intervention was successful. Not only did it increase overall use of contraception by 3 months, but it shifted that contraceptive use away from shorting-acting methods in favor of longer-acting methods, with high continuation and satisfaction rates and no adverse outcomes reported. We look forward to our 12-month outcomes to provide additional detail on longer-term continuation, satisfaction, and pregnancy rates as well as reach and effectiveness of our intervention from women’s perspectives.

## Data Availability

Data available on request due to privacy/ethical restrictions.
